# The possibilities of radiofrequency technology in the surgery of parenchimatous organs

**Published:** 2009

**Authors:** Ilic Miroslav, Milovancev Aleksandar, Koledin Milos, Gavrancic Brane, Raffay Violetta

**Affiliations:** * Institute for Lung Disease of Vojvodina, Clinic for Thoracic Surgery, Sremska Kamenica, Serbia; ** Municipal Institute for Emergency Medical Care, Novi Sad, Serbia

## Abstract

The aim of this paper is to determine the possibilities of modern radiofrequency (RF) technology and the usefulness of abdominal and thoracic parenchymatous organs in surgery. Investigation was made on 17 patients with 125 RF energy realized cycles (an average of 7,35 per one pt.) and the average time heating coagulative necrosis of 42,6 minutes (maximum to 80 minutes). There was one complication (pleural effusion) in a patient with RF treatment of 5 metastases colorectal carcinoma (MCRC) and synchronous right hemicolectomy. There were no other complications either to close or to distant to the organs. The urgent need of RF technology was in the case of a patient with iatrogenic rupture of spleen, treated by radiofrequency coagulation (RFC) with documented preservation of the whole organ. Most of the patients with MCRC (64%) were intraoperatively treated with a combination of radiofrequency ablation (RFA) and radiofrequency assisted resection (RFAR) of the liver with success in 95% of the cases. In the surgery of echinococcal liver cyst located deep, in the parenchyma, RFA were used for scolicidal purpose, and for hepatotomy. In the treatment of lung malignancies RF technology was reserved for nonsurgical candidates suffering from NSCLC, but also for surgical patients as a palliative measure in the treatment of local symptoms related to non-resectable primary and secondary tumors, presenting an aggressive tumor growth on the thoracic wall and the great vessels, with the possibility of reducing the number of explorative thoracotomy.

## Introduction

The application of heated coagulative necrosis, in other words, irreversible cells damage by heating due to denatured proteins and by using radiofrequency energy (RF), well known in human medicine as the ablation method for more then 20 years, lead to the loss of bilipid membrane strata and intra-cellular destruction of DNA, RNA, at 100ºC temperature [**1**]. Refining RF technology (energy generators and electrode needles), also by expanding the fields of usage in tumor processes of parenchyma organs, brought the change and innovations in intervention radiology and the strategy of surgery, these being the first among all the ablative techniques and all the resection techniques [**2**,**3**]. The aim of this research is to examine the possibilities of using radiofrequency technology in surgical treatment of diseases of parenchymatous organs located in the abdomen and the thorax.

## Material and methods

**Fig. 1:**
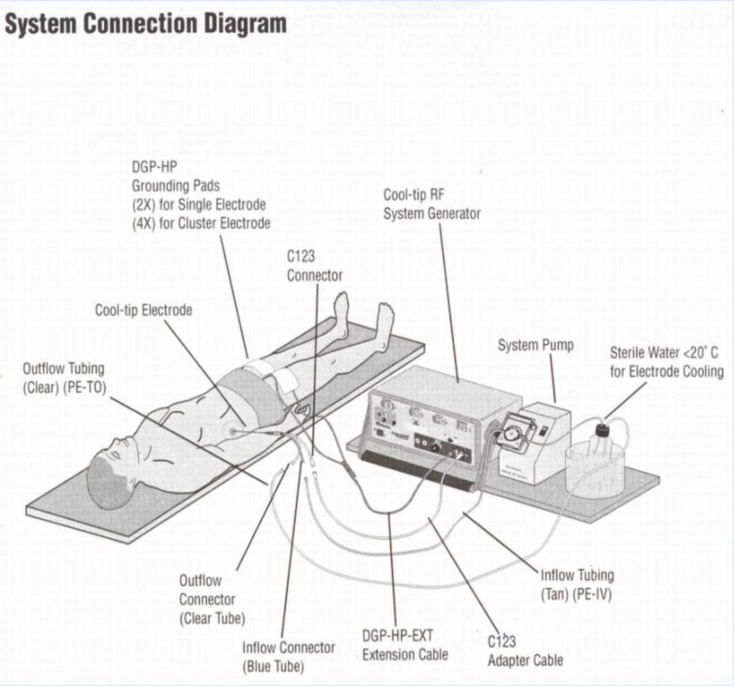
The system connection diagram

Research was performed in the Clinic of Thoracic Surgery of Lung Disease Institute in Sremska, Kamenica, from the beginning of 2005 untill the middle of 2006 (a 16 months period). The device used for tissue coagulation had 17G needles, with different lengths (10, 15 and 25cm), the diameter of the coagulation sphere was of 2 to 4cm, the “cold top” (Cool-tip RF Tissue Coagulation System, Valleylab, Tyco Healthcare Group LP, USA), a 200W generator and a pump with the possibility of moving through the 60mL/min electrode with additional reservoir for cold sterile water which refrigerates the top of the needle. In order to set the electrical circle between the uncoated top of the needle and the tissue of the parenchymatous organ (”positive” electrode) two (max. 4) ”negative” electrodes were set on the patient’s haunches (**Fig. 1**).

The system was set after the beginning of general anesthesia. The ”manual” system of RF energy release in all patients was used in case there was a possibility of reaching the temperature of 99ºC in the minimal time period. The requirement for a quick RF energy release was righteous by direct operative control (and by extra usage of diagnostic ultrasound) of the top of the needle, its position and coagulation process, presenting the option of multi-repeated cycle of RF energy emission and by needle position change, which corresponds to the surgical use of all system. Due to the fact that temperature between the needle and the tissue was measured continually, sound signal reports discontinued RF energy emission, in which case complete coagulation tissue necrosis was achieved (”radiofrequency ablation” – RFA). In case multi-repeated RF energy release was needed along with all sequences of coagulation necrosis (most frequently to present ”radiofrequency resection” – RFAR), a process that was repeated in the cycles of RF energy release, was needed to achieve a tumor resection with a different diameter.

Research included analyses of two data groups: those connected with the use of RF technology in the operating room (A) and the data about RF technology success in the treatment of pato-morphology substrate to parenchymatous organs of thorax and abdomen (B). The following data were registered: the existence of problems linked with the generator, needle, pump and RF energy emission process at all, number of applied RF cycles, duration of the procedure, possible tissue damage (close and distant), needle ”cool type” application to different lesions and parehchymatous organs, possibility of RF energy to stop the bleeding of the organs from abdomen and thorax, number of treated lesions in some organs (liver), prospective use other and additional ways to stop the bleeding (Pringels maneuver to liver, ligature to large blood vessels, and blood use) and also complete success of RFK, RFA and RFAR (with the applicance of intra-operative and post-operative morphology diagnostics). Data were processed by standard statistical methods.

## Results

Results in correlation with the application of RF technology in operating room (group A data):

1. A sum of 125 cycles of RF energy releases were applied in 17 patients (from 1 to 22, on average 7.35 cycles per patient), the period necessary to perform RF procedure was from 12 to 80 minutes (the average of RF procedure duration is 42.6 minutes). There were averagely 2.5 cycles for RFC procedures and for 2.5 cycles RFA as well. As far as RFAR 10 and the combination of RFA/RFAR are concerned there was an average of 12 cycles of RF energy release.

2. No technical problems related to the RF procedure were recorded during working, the procedure was performed on all patients electively or previously planned, or it was decided to be performed intra-operatively with the establishment of the whole system of RF, few minutes after the surgeon‘s request. 

Technical problems were not recorded with the "cool type" needle; it was easy to handle when accessing the patomorphology supstrate in all cases. 

3. There were no negative effects of RF energy to distant organs or tissues, intra-operatively and postoperatively.

4. The radiofrequency coagulation (RFC), as an unique procedure, was done in the cases of 2 patients (12%), radiofrequency ablation (RFA), in the cases of 4 patients (24%) – in two percutaneous RFA liver metastases leaded by US, and in two patients in open laparotomy, thoracotomy procedure. While radiofrequency assisted, resection (RFAR) of liver was performed in 5 patients (29%). Combinations of RFA and RFAR were done in 6 patients (35%);

5. RF technology was successfully applied on 17 patients in 13 cases as a predicted method of treatment (elective indication for the application of RF technology), in the case of two patients it was used to solve a bleeding problem which incidentally occurred during the operation. There was a decision to apply RF on the other two patients as a treatment of tumor; this decision was made intra-operatively (there were four patients with an urgent indication of RF technology application).

The indication area (group of information B) for the application of RF technology is presented in the **Table 1**.

**Table 1:** Indications for the application of the RF technology

Disease	Number of patients
Colorectal carcinoma metastases in liver	12
Echinococcus of the liver	1
Liver Adenoma	1
Iatrogenic spleen rupture	1
NSCLC /bleeding/	1
Osteosarkoma metastases in thorax	1

**Fig. 2:**
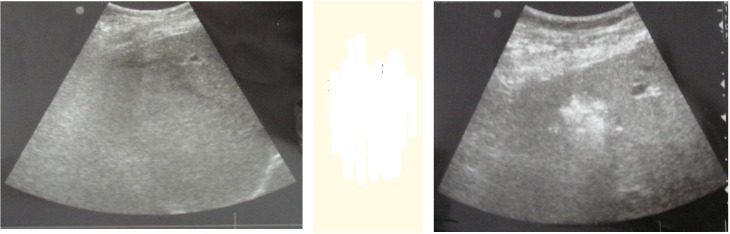
Percutanous US: MKRC before and after RFA

The group of patients with metastases of colorectal carcinoma (MCKRC) of liver (12) was diagnosed before the operation 22 MKRC. Two patients with solitary MKRC were treated with RFA in a percutanous way, and the process of monitoring was observed by ultra sound in the operation room (**Fig. 2**).

**Fig. 3:**
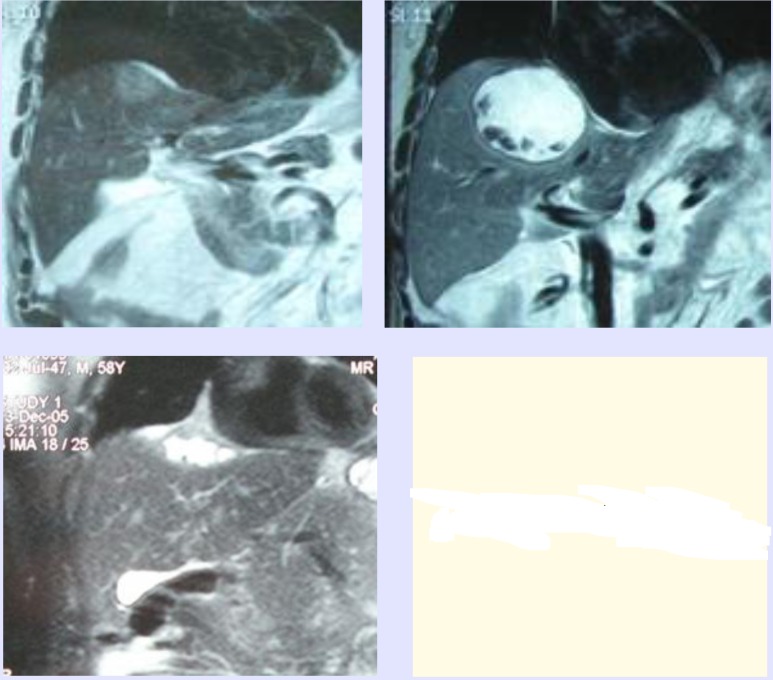
MRI of liver: MKRC before, immediately after RFAR and after 8 months

From 10 patients who were operated (laparotomy with the compulsory IOUS of liver), six had solitary MKRC (larger then 4cm) and all have been solved by RFAR (**Fig. 3**).

**Figure 4:**
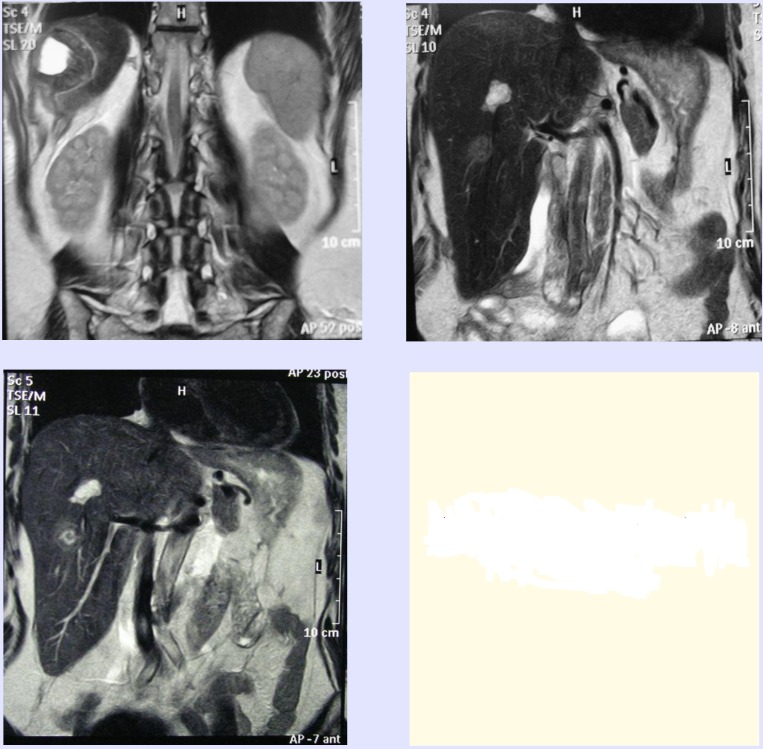
Postoperative MRI after RFAR with afterwards RFA of metastases and liver hemangioma

Multiple MKRCs were recorded on four patients and were solved according to the rule of RFAR (if they were on the surface of the organs), and RFA, if they were less than 4cm, or were situated in the depth of liver parenchima. There were two metastases which were solved by “deep” RFA under IOUS. There were five (according to the changes) MKRCs treated with the combination RFAS/RFA, (3 RFAR + 2 RFA) on one patient, where, except for the liver operation, there was another operation of primary colon transversum cancer, performed (extended right hemicolectomy) after initial chemotherapy. The case of this patient recorded the only complication of total treatment – right side pleural effusion treated by repeated pleural puncture. In postoperative MRI (performed on all patients suffering from MKRC) there was a fixed percent of success for the complete removal of MKRC by using RF technology: out of 22 cases of MKRC, 21 of them were successful - 95%. One patient suffering from multiple MKRC (where re-resection of liver was performed –RFAR and RFA) was performed a MRI examination and it was discovered that he had one unsolved metastases, situated in the depth of liver parenchyma (size 2cm) covered with liver hemangioma. It was successfully solved by percutaneous RFA (**Fig. 4**).

**Fig. 5:**
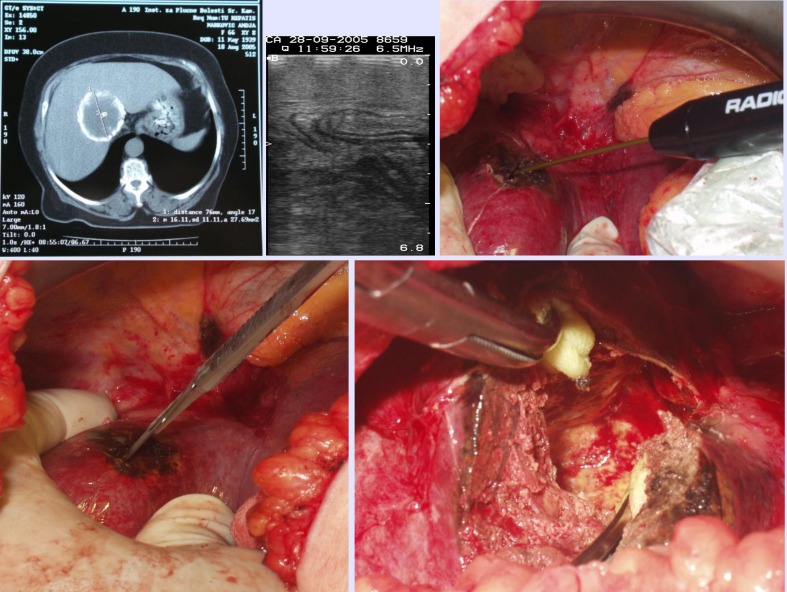
Pre-operative CT of echinococcal liver cyst, IOUS, RFK, hepatotomy without bleeding, cavity of ectocyst through the hepatotomy.

It is important to emphasize the fact that, in the application of RFAR of liver (actually the combination of RAFAR/RFA), neither Pringles maneuver, nor blood donation to the patient haven’t been used during the operation. Three patients with large exposed blood vessels were applied ligature, stitch-ligature of the larger blood and biliary ducts in liver hilus. Neither death outcomes nor postoperative complications appeared (except for the mentioned pleural effusion) and the patients had to stay in the hospital for only four days. One patient suffering from liver echinococcus, morphologically localized in the right lobe, between medial and right hepatic vein, vena cava, was performed a combination of RFA content of echinococcal cyst and RF hepatotomy, through which the cyst was opened and its contents were emptied (**Fig. 5**).

Urgent indications of RF technology were applied in correlation with the treatment of intra-operative bleeding, in the case of one patient with iatrogenic rupture of spleen during left adrenalectomy (patient with primary NSCLC). Moreover, a patient with NSCLC metastasis and bleeding from the infiltrated vertebra, after metastasectomy, was given the same treatment. The iatrogenic rupture of spleen had a length of 7cm, and the deepness of 2cm, presenting important bleeding. It was treated successfully by two cycles of RFK. So, post-operative observation of that complete preservation of the spleen and its functional status was reported during a 6 months’ period (**Fig. 6**)

**Figure:**
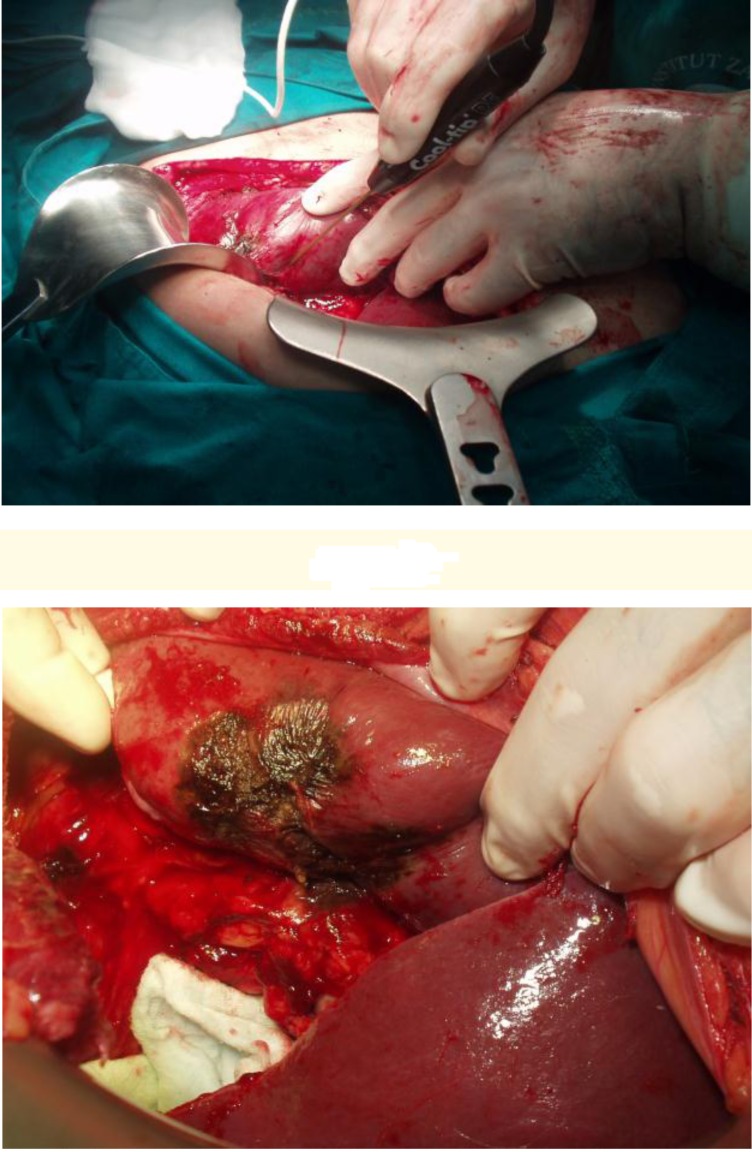


The liver adenoma, localized by morphology close to cholecyst, found during laparoscopic cholecystectomy, sized about 5cm, was solved by RFAR.

In a patient with osteosarcoma metastases, localized in the upper aperture of thorax, RFA was done, after the explorative thoracotomy and the estimated risk of resection have been established.

To sum up, the results of RF technology application in the stated indications, pointed out that success is great (approximately in all cases), and its application does not affect the other distant organs.

## Discussion

The conduction of high frequency alternating current electricity (more than 10kHz) through the tissue leads to an increase of temperature in that tissue, without muscle contraction or pain. Electrical area dislocate molecules in one direction, and then in other, causing ionic agitation, which by its movement leads to temperature increase in tissue. If electrodes (which have an electric area between them) are equal, ion movement will be equal. However, if one electrode is smaller, its close temperature will be higher, because ionic agitation in that place will be of higher “consistency“. The action of radiofrequency energy in clinical conditions depends on the interaction between electrode and tissue. Radiofrequency energy has been often used during the past few years to stop abnormal ways of heart conduction system. For that purpose electrodes and generators with low power (less than 50 W) were used. McGahan has developed the first generation of ablative mono-polar electrodes, during the ’90s and demonstrated that radiofrequency energy may cause heated coagulative necrosis of liver tissue, having the length of 10mm [**4**]. Then, the author presented this as being “radiofrequency electrocautery“. The invention of “inter - cooled needles“, monopolar electrodes with cold sterile fluid passing through its canal to the top of the needle, with the intention of widening the area of heat energy emission in tissue (to 3cm) and the generator escalation (to 200W), brought RF technology to its peak, being considered an important ablative handle. We used this kind of advanced equipment in our research. A high power generator, the process of multi heated-energy release to “target” tissue of parenchyma organ, raised a new question of safety with concern to this application in surgical conditions.8 cycles of heating emission per patient were noted in our material (in some cases 22 cycles), without consequences, observing manufacturers’ guidelines and setting up to 4 electrodes to patient’s haunches.

A complete period of RF emission was sometimes up to 80 minutes, with an average period of 42 minutes, without any technical damage of the needle, in other words without any problem of internal cooling. The problem of heat energy transmission to local organs [**5**] is mentioned in literature and was present in patients treated with RFA, by percutaneous application, while adhesion organs (colon, stomach etc.) were nearby. So, damage was done. In our case, the percutaneous electrode (two cases) set had a directly observable position through the nearest part of the front lateral abdomen wall (rib arch). All the other patients were treated by direct operative visualization of the needle’s top, but by IOUS use, which means that the needle’s position was in the center in order to observe the coagulative necrosis. Taking into account this approach, it is quite clear that surgical use of RF technology is reliable, especially to liver, if a request of understanding a well diagnosed ultrasonography (percutaneous and intraoperative) is made. The application of RF energy was first used in tumor ablation, “radiofrequency ablation“ RFA in liver. However, other parenchymal organs were started by the intervention radiologists, setting the refined electrode needle in the tissue percutaneously, being led by ultrasonography or CT [**6**]. Although success of RFA was superior compared to some other ablative techniques, such as percutaneous ethanol injection (PEI) in hepatocellular carcinoma [**2**], there is a problem with the area of RFA for parenchymatous organs, due to two reasons: the first one refers to the presence or the absence of the extrahepatic disease and its assessment, and the other one refers to the technical limitations of diameter for possible RFA. Due to these reasons surgical treatment still remains a standard to solve focal lesions of liver and of other parenchymatous organs too [**7**].

Considering the fact that it is possible to get a good insight into the extent and expansion of liver changes by a surgical (laparoscopic) exploration, it has soon become clear that RFA needle can be applied to laparoscopic operations, that is, as a part of open surgical procedures (laparotomy, thoracotomy). In this way, a surgeon is introduced to RF technology and its practical application [**8**]. Most of RF procedures in our material, except for the percutaneous application of the RF electrode, have been performed in open surgeries and only one in a laparoscopic surgery. The reason for a small number of the laparoscopic applications of the RF needle lies in the fact that the largest number of patients had already had laparotomy due to treating metastases of the earlier colorectal cancer. In addition, a large number of intra abdominal growths have been expected, which made the laparoscopic RF procedure even more difficult. An incidental detection of the periphery liver adenoma (during a laparoscopic cholecystectomy) has been used for the laparoscopic application of the RF needle (**Chart 1**).

The RF technology in our material has been used not only as an elective procedure but also as an emergency one. In both cases, it was extremely important to quickly set up the apparatus for operations on iatrogenic spleen ruptures, stopping abundant bleeding and minor hemorrhage from the infiltration of NSCLC spinal column. In literature, there are well known data on practical application of the RF technology in spleen surgery, including resections and biopsies of this highly rich-blood abdominal parenchymatose organ, whose “conventional” surgery has been mostly restricted to splenectomy [**9**]. The success of stopping the bleeding in both cases, one of them involving the possibility of splenectomy and achieving complete salvage, shows the need for the presence of the RF technology in all operating theatres performing elective and emergency surgery, as well as introducing a large circle of surgeons to its performance and possibilities.

In our experience, the RF technology has been used with the largest number of patients (64%) as a resection procedure or as a combination of RFAR and RFA. The possibility of a “bloodless” transection of the liver parenchyma without the use of Pringle`s maneuver appeared since the article on liver resection by thermal coagulated necrosis using the RF technology was published by Habib in 2002 [**2**], and later by other authors [**10**]. The technique includes causing metastasis to the surrounding coagulated necrosis and then using a scalpel to dissect a part of the tissue. On such an occasion, and in the case of a possible bleeding, repositioning of the electrode for the purpose of additional coagulation (RFK) is used. This way, 10 patients were successfully treated with 14 metastases of the colorectal cancer alltogether. The RFK/RFA process in the tissue takes place after the evaporation of the cell fluid followed by bubbles of gas (nitrogen) coming from the tissue. If the process is followed by an ultrasound diagnose, the usual echogenicity of the change alters towards hyperechogenicity, considering the fact that the gas occupies the whole diameter of the coagulated sphere. The proximity of blood vessels, through which blood flows, that is, the border of the tissues with different electric features causes a halt in the thermal coagulated process, so that the danger of damaging bigger blood vessels is very small. The RFA process is basically limited to a local pathomorphic substrate within the parenchymatose organ (**Figure 4**). If the RFA takes place in the liver, and a bigger sphere of ablation is desirable, it is possible to stop blood circulation in the hilum, just like putting a saline solution into the “target” tissue and thus make the ablative sphere bigger. We did not apply Pringle`s maneuver on our material nor did we add fluid into MKRC. No intra-operative blood supply had been given, not even during bigger resections. Only once during the RFAR process did we use ligatures for big biliary structures in the liver hilum. Considering the danger of high temperatures in the hilum, mainly in large biliary structures, we have worked out a methodology of intrahepatic biliary rinsing with a cold solution. This way, large biliary structures are prevented from thermal damage. There was no need for such a maneuver in our material because of MKRCS.

A combination of safe (in relation to bleeding and metastasectomy) RFA and RFAR makes it possible to broaden indications for “gradual” eradication of metastases of colorectal cancer, with the possibility of re-resecting liver [**11**]. We had 2 patients who underwent re-resection in our material, one of whom we performed an additional RFA upon. (**Fig. 4**). In the case of the patient who underwent right hemicolectomy, the combination of RFAR/RFA successfully treated 5 synchronous MKRCs. Special attention in the surgery of MKRCs was paid to adequate margins, which should have been less than 1cm from the metastasis. We have ascertained in our work the success of RFAR and RFA in accomplishing this oncology task (**Fig. 3**).

The RFA technology has also been used during a surgical procedure on echinococcal liver cyst, localized in the corner between the right and the middle hepatic vein, with a double role: by “cooking” the content of the cyst itself for the purpose of decreasing the cyst's vitality, as well as the “bloodless” hepatotomy. Since Brunette's report in 2001 [**12**], the RFA of echinococcal liver cyst has been recognized in the treatment of this disease. In our case of echinococcal liver cyst, where there was a crumpled vital germinal membrane present, the content of the cyst had been “cooked” for 12 minutes under IOUS.

In the surgery of echinococcus, many scolicid agents have been applied, but an ideal scolicid solution has not been found yet. It is possible that the thermal damaging of cysts is a new approach in the radical treatment of this disease. However, to draw such conclusion, a bigger surgical experience and a rational position of RFA are needed in the problem-solving surgery of echinococcal disease, especially from the point of view of different stages of echinococcal cyst. Anyways, parasitic focal changes in the liver can be treated intraoperatively by RFA. 

One of our patients in our material was performed the RFA through thoracotomy for the purpose of treating the infiltration of the upper pectoral aperture with metastases of osteosarcoma. When talking about the application of the RFA technology in treating intrathorocal pathological processes, usually two main groups of indications are mentioned: a) the RFA of lung malignancies with the intention of definite treatment; there are patients who are not candidates for a surgery due to comorboid condition, as a contraindication of surgical treatment (bad cardiopulmonary reserves) and patients who refuse to have a surgery b) the RFA as a palliative treatment of lung malignancies (for the purpose of tumor reduction before chemotherapy or controlling the symptoms due to aggressive tumor growth, that is the infiltration of bone structures) and, in relapse of tumors, patients who are not in condition to have another radiotherapy or surgery [**13**]. Surgeries represent the standard of NSCLC curative treatments [**14**]. Of all discovered NSCLCs, and there are about 80% of newly discovered lung malignancies, only one third can be surgically treated [**15**]. That is why new alternative approaches are being intensively considered, those that can lead to the destruction of tumors, or even complete eradication or as a complementary treatment or as a treatment that makes already tried ways better. The RFA technology in the NSCLC treatments, described on an animal model of lungs in 1995 [**16**] and on human lungs in 2000 [**17**] represents, mainly, a minimally invasive, non-operative way in the hands of radiologists along with the CT, which is done in specialized centers as an ambulant procedure with minimal complications on patients who are not candidates for surgeries. Associated at first to its application in operating rooms or to patients who have been already put through operation, RF technology was successfully used for inoperable osteosarcoma metastases and for vertebral bleeding after metastases were removed by NSCLC. These results indicate that in patients with advanced intrathorax malignancy, the application of RF palliative treatment is possible and will have an opposite end with explorative thoracotomy.

## Conclusion

Modern RF technology which uses mono-polar “internal cooling“ electrodes in the shape of thin needles and high power generators (200W), allows fast and effective achievement of heat coagulative necrosis with different diameter of ablative sphere (from 1 to 3cm), depending on the way and the aim that can be found in clinical situations applied as: radiofrequency coagulation (RFC), radiofrequency ablation (RFA) and radiofrequency resection (RFAR). The process of RF energy emission securely proceeds to local and distant organs, in surgical conditions, while direct visual control of the top of the electrode, practically without limitations, exposes the causes of necrosis coagulation (to final lasting up to 80 minutes) in order to treat some of the focal lesions of parenchymatous organs. The needle (internal cooled electrode) from 17G (to 19G ) is especially convenient for intra operative manipulation, for its right positioning in focal modification of liver and the observation of hyper thermo coagulation process, with gas release. It is important for the surgeon to understand the ultrasonography diagnostics, especially the intraoperative ultrasound (IOUS). The use of RF technology in surgery of the liver and spleen take for granted the combination of RF ablation, RF coagulation and RF resection (in two thirds of the cases). RF technology is very successful in the treatment of abdominal organs and is used in urgent operations. This is very important to the spleen with imperative attitude about its preservation, so, the presence of this equipment is needed in all operating rooms, with required acquaintance of the possibilities and techniques of RFC, RFA and RFAR to surgeons. RF technology application in treatment of colorectal carcinoma metastases, with the observance of standard criteria, is useful in all patients, with the combination of RF ablation (operative and percutaneous) and RF resection, with minimal complications, practically doing “no blood“ transsection of liver parenchyma, without Pringel maneuver. It is possible to have rational RF use in echinococcal liver disease too. RF technology in lung malignancy treatment, as first reserved for patients with NSCLC which are not candidates for surgery treatment, can contribute to palliative treatment of local unresected and furthered primary and metastases tumors in patients, which above all infiltrates thorax’s wall and large blood vessels, with decreased numbers of “explorative“ thoracotomy.
